# Iodized Salt Sales in the United States

**DOI:** 10.3390/nu7031691

**Published:** 2015-03-10

**Authors:** Joyce Maalouf, Jessica Barron, Janelle P. Gunn, Keming Yuan, Cria G. Perrine, Mary E. Cogswell

**Affiliations:** 1 Division for Heart Disease and Stroke Prevention, National Center for Chronic Disease Prevention and Health Promotion, Centers for Disease Control and Prevention, Atlanta, GA 30341, USA; E-Mails: JPeralezGunn@cdc.gov (J.P.G.); KYuan@cdc.gov (K.Y.); MCogswell@cdc.gov (M.E.C.); 2 IHRC, Inc., Atlanta, GA 30346, USA; 3 Oak Ridge Institute for Science and Education, Oak Ridge, TN 37831, USA; E-Mail: jessylbarron@gmail.com; 4 Division of Nutrition, Physical Activity, and Obesity, National Center for Chronic Disease Prevention and Health Promotion, Centers for Disease Control and Prevention, Atlanta, GA 30341, USA; E-Mail: CPerrine@cdc.gov

**Keywords:** salt, sodium, iodine, sales, United States

## Abstract

Iodized salt has been an important source of dietary iodine, a trace element important for regulating human growth, development, and metabolic functions. This analysis identified iodized table salt sales as a percentage of retail salt sales using Nielsen ScanTrack. We identified 1117 salt products, including 701 salt blends and 416 other salt products, 57 of which were iodized. When weighted by sales volume in ounces or per item, 53% contained iodized salt. These findings may provide a baseline for future monitoring of sales of iodized salt.

## 1. Introduction

Adequate intake of iodine, a trace element, plays a key role in regulating human growth, development, and metabolic functions. It is estimated that, worldwide in 2011, 1.88 billion people were iodine deficient [1,2]. Iodine deficiency can result in goiter, neurocognitive impairment, hyperthyroidism, and hypothyroidism [3]. During pregnancy, severe iodine deficiency can cause cretinism, congenital anomalies, and increased neonatal and infant mortality [2], with recent evidence from observational studies suggesting mild to moderate deficiency during pregnancy can lead to reduced IQ and educational achievement among offspring [4,5]. While iodine status is sufficient for most Americans, certain subsets of the population, including pregnant women, may be at risk for mild to moderate iodine deficiency [6,7,8]. Iodizing salt is a global public health strategy for addressing iodine deficiency [9], yet limited data exist on the proportion of salt that is iodized in the United States.

Historically, iodized salt has been an important source of dietary iodine for Americans; however, not all regular salt is iodized, nor are specialty salts, such as sea salt and kosher salt [10]. Fortification of salt with iodine in the United States is voluntary [11]. A past estimate indicates that approximately 70% of salt sold to US households is iodized though the method of determining this estimate is unclear [12]. Using Nielsen sales data, this brief provides information on the proportion of iodized and non-iodized table salt sales at the retail level.

## 2. Methods

Nielsen ScanTrack (The Nielsen Co, New York, NY, USA) [13] data from 2009 was used to identify salt products sold in the United States. Nielsen collects information from a stratified probability sample of scanner-equipped chain and large independent grocery stores with estimated total annual sales ≥$2 million. Sales data are aggregated and not linked to individuals [13,14]. A total of 1117 salt products were identified using 2 Nielsen grocery modules. Salt products were initially categorized as iodized using the term “IDZD” in the Nielsen product description (*n* = 67). Ingredient and label details were obtained using the manufacturer’s and others’ websites, following a standard Internet search protocol [15]. Salt products were considered to be iodized if the front of the label or the product name specified it and if iodine was reported on the back in either the nutrition facts panel or the ingredients. Two nutritionists categorized the salt products as iodized or non-iodized and a third resolved any differences.

## 3. Results

In total, 1117 salt products were identified, of which 701 were salt blends (e.g., garlic salt) comprising 16% of the total sales volume of salt products (Table 1). The number of salt products can include more than 1 product from the same brand (e.g., different size containers). Of the remaining 416 salt products, 57 were iodized based on available label information. When weighted by sales volume (in ounces or by each sold), 53% of salt sold (excluding salt blends) was iodized. Of the 47% of non-iodized salt sold, about 68% was regular salt, 19% kosher salt, and 12% sea salt.

**Table 1:** Proportion of Total Salt Sales by Type of Salt, 2009 ^a^.

Salt Type	No. in Sample ^b^	Total Sales (%) ^c^	Sales Excluding Salt Blends ^d^ (%) ^e^
**Iodized salt**			
Regular/plain table salt	35	42.2	50.1
Salt & black pepper shaker packet ^f^	13	1.5	1.8
Sea salt	9	1.1	1.3
Subtotal	57	44.7	53.2
**Non-iodized salt**			
Regular/plain table salt	46	26.8	31.8
Salt & black pepper shaker packet ^f^	22	0.04	0.1
Sea salt	14	7.6	5.5
Kosher salt	169	4.7	9.0
Artisan/gourmet/finishing salt	108	0.3	0.4
Subtotal	359	39.4	46.8
Total (excluding salt blends ^d^)	416	84.1	100
**Salt blends ^d^**	701	15.9	
Total (including salt blends)	1117	100	

^a^ Nielsen ScanTrack data; ^b^ The number of salt products can include more than 1 product from the same brand (e.g., different size containers); ^c^ Percent of total salt sales weighted by volume of sales; ^d^ Salt blends (e.g., garlic salt, onion salt, seasoning salt); ^e^ Percent of total salt sales excluding salt blends weighted by volume of sales; ^f^ Salt in salt and pepper shakers sold as packet were labeled as iodized or non-iodized salt.

## 4. Discussion

This analysis identified that only 53% of table salt sold at the retail level in the United States is iodized. The vast majority of sodium intake is estimated to come from packaged and restaurant foods. It is estimated that only 11%–12% comes from salt added to the table or salt added during home cooking [16,17]. To our knowledge, this is the first study to report on the proportion of iodized and non-iodized salt sold at the retail level in the United States. Nielsen ScanTrack data include many products within a given category such as salt. ScanTrack data also include major food retailers in operation across most of the US market. However, sales data from Nielsen ScanTrack do not include warehouse stores (e.g., Walmart and Costco), retailers with sales <$2 million, or non-UPC-coded products, and not all places where salt can be purchased were tracked. These findings may provide a baseline for future monitoring of sales of iodized salt in major food retailers in the United States. Tracking the sales of iodized salt over time will help describe potential shifts in the U.S. market share in response to ongoing efforts to reduce salt intake and ensure iodine sufficiency [18,19]. Increasing the proportion of regular salt that is iodized may help improve iodine intakes among pregnant women in the US; however, public health interventions that aim to ensure iodine sufficiency among pregnant and reproductive-age women must also be aware of the potential for excess intake of iodine in other populations [20].

## Human Participation Protection

Institutional review board approval was not needed because it did not involve human participants.
